# Loss of Dickkopf 3 Promotes the Tumorigenesis of Basal Breast Cancer

**DOI:** 10.1371/journal.pone.0160077

**Published:** 2016-07-28

**Authors:** Eva Lorsy, Aylin Sophie Topuz, Cordelia Geisler, Sarah Stahl, Stefan Garczyk, Saskia von Stillfried, Mareike Hoss, Oleg Gluz, Arndt Hartmann, Ruth Knüchel, Edgar Dahl

**Affiliations:** 1 Molecular Oncology Group, Institute of Pathology, Medical Faculty of the RWTH Aachen University, Aachen, Germany; 2 Electron Microscopy Facility, Medical Faculty of the RWTH Aachen University, Aachen, Germany; 3 West German Study Group, Breast Center Niederrhein, Bethesda Hospital, Monchengladbach, Germany; 4 Institute of Pathology, University Hospital Friedrich-Alexander University Erlangen-Nuremberg, Erlangen, Germany; Florida International University, UNITED STATES

## Abstract

Dickkopf 3 (DKK3) has been associated with tumor suppression of various tumor entities including breast cancer. However, the functional impact of DKK3 on the tumorigenesis of distinct molecular breast cancer subtypes has not been considered so far. Therefore, we initiated a study analyzing the subtype-specific DKK3 expression pattern as well as its prognostic and functional impact with respect to breast cancer subtypes. Based on three independent tissue cohorts including one *in silico* dataset (n = 30, n = 463 and n = 791) we observed a clear down-regulation of DKK3 expression in breast cancer samples compared to healthy breast tissue controls on mRNA and protein level. Interestingly, most abundant reduction of DKK3 expression was detected in the highly aggressive basal breast cancer subtype. Analyzing a large *in silico* dataset comprising 3,554 cases showed that low *DKK3* mRNA expression was significantly associated with reduced recurrence free survival (RFS) of luminal and basal-like breast cancer cases. Functionally, DKK3 re-expression in human breast cancer cell lines led to suppression of cell growth possibly mediated by up-regulation of apoptosis in basal-like but not in luminal-like breast cancer cell lines. Moreover, ectopic DKK3 expression in mesenchymal basal breast cancer cells resulted in partial restoration of epithelial cell morphology which was molecularly supported by higher expression of epithelial markers like E-Cadherin and down-regulation of mesenchymal markers such as Snail 1. Hence, we provide evidence that down-regulation of DKK3 especially promotes tumorigenesis of the aggressive basal breast cancer subtype. Further studies decoding the underlying molecular mechanisms of DKK3-mediated effects may help to identify novel targeted therapies for this clinically highly relevant breast cancer subtype.

## Introduction

Breast cancer is the most frequently diagnosed cancer and the leading cause of cancer deaths among females worldwide [1]. Nevertheless, the clinical outcomes differ between the distinct biological breast cancer subtypes [2]. Based on gene expression profiling breast cancer is categorized into five intrinsic subgroups: luminal A (mostly estrogen-receptor (ER)-positive and histologically low-grade), luminal B (also predominantly ER-positive and often high-grade), HER2-enriched (often show amplification and high expression of the *ERBB2* gene), basal-like (mostly corresponding to ER-negative, progesterone-receptor (PR)–negative and HER2-negative tumors thus triple negative breast cancer (TNBC)) and normal-like cases, which clinical relevance is still controversial [3–6]. While luminal A breast carcinomas are associated with good prognosis, breast tumors of luminal B, HER2-enriched and basal-like subtypes are related to unfavorable clinical outcomes [4, 7]. Furthermore, breast cancer subgroups differ in their responses to therapy. Whereas luminal tumors are sensitive to endocrine therapy as they are estrogen receptor positive, HER2-enriched carcinomas can be treated with Trastuzumab, an anti-HER2 antibody [3]. However, treatment of basal/triple negative tumors lacks in alternatives to chemotherapy underlining the urgent need to develop novel targeted, less toxic therapies for this breast cancer subtype [8]. To achieve this goal, further understanding of molecular alterations underlying the subtype-specific carcinogenesis is required.

WNT-signaling regulates a range of physiological processes including embryonic development, cell proliferation, stem cell maintenance, epithelial—mesenchymal interactions but is also linked to human carcinogenesis [9–11]. Aberrant activation of this pathway by genetic and epigenetic alterations is a common event observed in several human tumor entities including breast cancer [10–12]. In tumor cells expression of WNT-antagonists like members of the secreted frizzled-related protein (SFRP) and the Dickkopf (DKK) family is frequently downregulated via epigenetic modifications [13–15]. The DKK family comprises four main members DKK1-4 and a unique DKK3-related protein named soggy (DKKL1). DKK proteins are secreted proteins which contain two conserved cysteine-rich domains and an N-terminal signal peptide [16, 17]. DKK1, 2 and 4 negatively regulate canonical WNT/β-catenin signaling in vertebrate cells by interacting with the co-receptors LRP5/6 and KRM1/2 [18, 19]. Nevertheless, DKK3 seems to have a divergent function as it neither binds LRP5/6 nor KRM1/2 [18, 20]. Its precise role in inhibiting WNT-signaling as well as involved receptors and the downstream signaling cascade are still uncertain [21].

First evidence for a potential relevance of DKK3 in tumorigenesis was given by the finding that DKK3 expression is decreased in immortalized cell lines, wherefore the gene is also named *REIC* (Reduced Expression in Immortalized Cells) [22]. Subsequently, loss of DKK3 expression mainly mediated by promoter-hypermethylation was demonstrated in the majority of tumor entities including breast cancer [21, 23]. Furthermore, it could be shown that DKK3 affects tumor cell growth due to induction of apoptosis in mammary carcinomas and several other cancer types and might be involved in the inhibition of epithelial-to-mesenchymal transition (EMT), indicating a role for DKK3 as a tumor suppressor gene [21, 24–26]. However, the biological function of DKK3 in the intrinsic breast cancer subtypes has not been analyzed so far.

In the present study we performed a comprehensive subtype-specific expression analysis of DKK3 in human breast cancer for the first time revealing pronounced loss of DKK3 in basal-type breast carcinomas. Moreover, we demonstrated strong growth suppressive effects mediated by DKK3 in basal-like breast cancer cells indicating a prominent role for DKK3 especially in the tumorigenesis of this clinically highly relevant breast cancer subtype.

## Material and Methods

### Cell lines and stable transfection

The human breast cancer cell lines MDA-MB-436, MDA-MB-231, MDA-MB-453 and MCF-7 were originally obtained from the American Type Culture Collection (Rockville, MD, US) and cultured under recommended conditions. Cell lines were regularly tested for mycoplasma infection using the PCR-based Venor^®^ GeM Mycoplasma Detection Kit (Minerva Biolabs, Berlin, Germany). Transfections were performed using FuGene HD Transfection Reagent (Roche, Mannheim, Germany) following the manufacturer's instructions. Either the pcDNA3.1/V5-His-TOPO vector construct containing the full-length human *DKK3* cDNA [27] or the pcDNA3.1/V5-His-TOPO empty vector (Invitrogen, Carlsbad, CA, USA) as control were used. Selection of stable *DKK3* and empty vector single-cell clones was achieved by culturing MDA-MB-436, MDA-MB-231, MDA-MB-453 and MCF-7 cells in complete culture medium containing 0.4 mg/mL, 0.7 mg/mL, 0.75 mg/mL and 0.5 mg/mL G418 (Life Technologies, Darmstadt, Germany) respectively for at least two weeks to ensure genomic cDNA integration. Afterwards, isolated clones were analyzed by both real-time PCR and western blotting for expression of DKK3.

### Cryoconserved clinical specimens

Cryoconserved tumor and normal breast tissue samples analyzed in this study were obtained from the tumor bank of the RWTH centralized biomaterial bank (cBMB, http://www.cbmb.rwth-aachen.de). All patients gave written informed consent for retention and analysis of their tissue for research purposes according to local Institutional Review Board (IRB)-approved protocols (approval no. EK-206/09) of the Medical Faculty at RWTH Aachen University. After surgery, tumor material was immediately snap-frozen in liquid nitrogen. Sections stained with hematoxylin and eosin, were prepared for assessing the percentage of tumor and normal epithelial cells, respectively. Only cryoconserved tumor samples containing more than 70% tumor cells, and normal samples containing at least 30% epithelial cells, were selected for RNA analysis. Tumor samples were classified by subtypes, i.e. “luminal”, “HER2-enriched” and “triple-negative breast cancer” (TNBC) [7, 28] based on immunohistochemistry (IHC) and fluorescence *in situ* hybridization (FISH) data for estrogen receptor (ER), progesterone receptor (PR) and human epidermal growth factor receptor 2 (HER2). Patient characteristics for fresh frozen samples are shown in the supplements (see S1 Table).

### Formalin-fixed, paraffin-embedded (FFPE) clinical specimens

Formalin-fixed, paraffin-embedded (FFPE) normal breast tissue samples analyzed in this study were obtained from the tumor bank of the RWTH cBMB. All patients gave written informed consent for retention and analysis of their tissue for research purposes according to local IRB-approved protocols (approval no. EK-206/09) of the Medical Faculty at RWTH Aachen University. Tumor samples were analyzed using a large tissue microarray (TMA) described previously [29, 30]. The TMA comprised 772 tumor tissues. 463 cases were included in the current study depending on analyzable DKK3 protein expression as well as available information about ER-, PR- and HER2-status for subtype-classification as described above. DKK3 protein expression was assessed according to an adapted immunoreactive score (IRS) developed by Remmele and Stegner [31]. Clinico-pathologic variables of breast cancer cases used in this study are summarized in S2 Table.

### TCGA patients’ data set

Raw IlluminaHiSeq expression data for *DKK3* as well as the corresponding clinical data of the primary breast cancer tissues (n = 791) and solid normal tissues analyzed (n = 113), were assessed using an independent and public data set from The Cancer Genome Atlas (TCGA) [32]. Using sample IDs (see S3 Table), the *DKK3* expression data of breast cancer specimens can be downloaded at the cBio Cancer Genomics Portal (http://www.cbioportal.org) [33], whereas the corresponding clinical data are available at *The Cancer Genome Atlas* data portal (https://tcga-data.nci.nih.gov/tcga/tcgaDownload.jsp). Tumor samples were stratified into subtypes by using the PAM50 gene signature [5]. Clinico-pathologic variables of breast cancer cases used in this study are summarized in S4 Table.

### Kaplan-Meier Plotter online tool

The potential prognostic impact of DKK3 in human breast cancer was analyzed using the Kaplan-Meier Plotter online tool (http://kmplot.com/analysis/), a database that integrates gene expression data and clinical data [34]. Employing Affymetrix microarray expression and corresponding survival data of the Kaplan-Meier Plotter portal, univariate survival curves over a time period of 120 months were calculated. For the analysis of recurrence-free survival (RFS) in relation to *DKK3* expression, data for 3,554 breast cancer patients were available. Survival curves were calculated considering all breast cancer cases as well as after stratifying the cohort by breast cancer subtype.

### RNA extraction and Semi-quantitative real-time PCR

Total RNA from cryoconserved tissues (20 μm^3^ each) was isolated using the standard procedure for TRIzol^®^ (Invitrogen, Carlsbad, CA, USA) RNA extraction. Extracted RNA was quantified using the NanoDrop ND1000 spectrophotometer (Thermo Scientific, Waltham, MA, USA). Subsequently, cDNA was synthesized using 1 μg of total RNA and the Reverse Transcription System (Promega, Madison, WI, USA) according to the manufacturer’s instructions. The IQ5-real-time PCR Detection System (Bio-Rad Laboratories, Munich, Germany) was used as described previously [35]. Gene-specific primer sets were designed by using Primer3web software (version 4.0.0). All reactions were performed in triplicates including negative controls without cDNA. Specificity of amplification products was confirmed by size estimation on agarose gels and melt curve analysis. Obtained data were analyzed using the comparative Ct (threshold cycle) method. Complete reaction conditions, primer sequences and lengths of amplicons are listed in S5 Table.

### Western blotting

Secreted proteins in the cell supernatant of transfected human breast cancer cells were concentrated by precipitation with 4 M ammonium sulfate overnight at 4°C. Simultaneously total cell protein lysates were obtained by sonification of the associated cells. Proteins were heat denatured (5 min, 95°C) and in each case approximately 30 μg of protein was loaded on 4–12% gradient gels (NuPAGE; Invitrogen) and then transferred onto 0.2 μm PVDF membranes (Whatman, Dassel, Germany) (1 h, 100 V) for immunodetection. Blots were blocked in TRIS-buffered saline (TBS) containing 0.1% Tween-20 (TBS-T) and 5% non-fat dry milk (Merck, Darmstadt, Germany) for 1 h at room temperature. Blocked blots were then incubated with the primary antibody overnight at 4°C, diluted in blocking solution containing 5% non-fat dry milk. The following primary antibodies were used: DKK3 (AF1118, R&D Systems, Minneapolis, MN), β-actin (A5441, Sigma-Aldrich, Deisenhofen, Germany). After washing three times (TBS + 0.1% Tween-20), blots were incubated with horseradish peroxidase-conjugated secondary antibodies (DAKO, Glostrup, Denmark) diluted in blocking solution containing 5% non-fat dry milk for 1 h at room temperature. After washing three times (TBS + 0.1% Tween-20), antibody detection was accomplished with Pierce ECL Western Blotting Substrate (Thermo Scientific, Rockford, IL, USA).

### Immunohistochemistry

Immunohistochemical analysis was carried out according to the manufacturer’s instructions (Vectastain PK-4005, Vector Laboratories, Burlingame, CA). Heat-induced epitope retrieval was performed in 10mM citrate buffer (pH 6.0) for 30 minutes using a water bath (98°C). FFPE sections (3 μm) were incubated for 40 minutes with a DKK3-specific antibody (1:20; AF1118, R&D Systems, Minneapolis, MN).

### Cell growth assay

Increase of cell number was recorded for MDA-MB-436, MDA-MB-231, MDA-MB-453 and MCF-7 *DKK3* and mock clones for 96 h. 3x10^5^ cells were seeded in six-well culture plates and cultivated for 96 h (20% O_2_, 5% CO_2_, 37°C). Thereafter the cell number was determined with the *CASY*^®^ *Cell Counter and Analyzer* (OLS OMNI Life Science, Bremen). Experiments were performed in triplicate.

### Apoptosis assay

Activity of the effector caspases 3 and 7 in stable MDA-MB-436, MDA-MB-231, MDA-MB-453 and MCF-7 *DKK3* and mock clones, as indicator of apoptosis, was determined by using the *Apo-One*^®^ *Homogeneous Caspase-3/7 Assay* (Promega, Mannheim, Germany) according to the manufacturer’s instructions. Briefly, cells (2×10^4^) were seeded in 96-well culture plates and incubated overnight (20% O_2_, 5% CO_2_, 37°C). Afterwards, staurosporine (final concentration 1 μM, Sigma-Aldrich, Deisenhofen, Germany) was added to induce apoptosis. After 24 h, lysis/substrate buffer was added leading to cleavage of the contained profluorescent caspase substrate Z-DEVD-R110 to create fluorescent rhodamine 110. The fluorescence signal, proportional to caspase 3/7 activity, was quantified by using an ELISA plate reader (excitation: λ = 499 nm; emission: λ = 521 nm). Experiments were performed in triplicate.

### Cell-matrix adhesion assay

Cell-matrix adhesion was assessed by coating six-well culture plates with 9.5 μg/ml *Matrigel* Basement Membrane Matrix (BD Bioscience, Heidelberg, Germany). MDA-MB-436 as well as MDA-MB-231 *DKK3* and mock clones (5×10^5^ cells/well) were plated, incubated for 90 min (20% O_2_, 5% CO_2_, 37°C) and gently washed three times with phosphate-buffered saline. Attached cells were fixed with 70% ethanol (10 min) and stained with 0.1% crystal violet solution (20 min). Cells were washed thoroughly with water and dried overnight. The dye was dissolved in 0.02% Triton X-100 in 100% isopropanol and carried over into a 96-well plate to measure the optical density at 590 nm. Experiments were performed in triplicate.

### Scanning electron microscopy

To prepare MDA-MB-436 *DKK3* and mock clones for scanning electron microscopy (SEM) cells were incubated on glass plates for 72 h under expansion conditions. Afterwards, cells were fixed in 3% glutaraldehyde (Agar Scientific, Wetzlar, Germany) for at least 4 h at room temperature, rinsed with 0.1 M sodium phosphate buffer (Merck, Darmstadt, Germany) and dehydrated by incubating consecutively in ascending ethanol series (30, 50, 70 and 90%) with a final incubation in 100% ethanol for 10 min. Last step has been repeated 3 times. Final drying step was performed with hexamethyldisilazane (Sigma-Aldrich, Steinheim, Germany) incubation for 10 min. Dried cells were sputter coated with a 10 nm gold palladium layer (sputter coater EM SCD500, Leica, Wetzlar, Germany). Samples were analyzed using an environmental scanning electron microscope (ESEM XL 30 FEG, FEI, Eindhoven, the Netherlands) in a high vacuum environment using acceleration voltage of 10 kV.

### Statistical analysis

Statistical packages SPSS 20.0 (SPSS, Chicago, IL, USA) and GraphPad Prism 5.0 (GraphPad Software, La Jolla, CA, USA) were applied for data analysis. To compare two groups, the non-parametric Mann-Whitney *U*-test and for comparison of more than two groups the Kruskal-Wallis test was used. Differences with a P-value < 0.05 were defined to be significant. The Fisher’s exact test was applied in order to correlate clinico-pathological parameters with *DKK3* mRNA expression. To determine the prognostic value of *DKK3* mRNA, univariate Kaplan-Meier survival analysis was performed using the Kaplan Meier Plotter data set [34].

## Results

### DKK3 expression is reduced in human breast cancer

**Fig 1:**
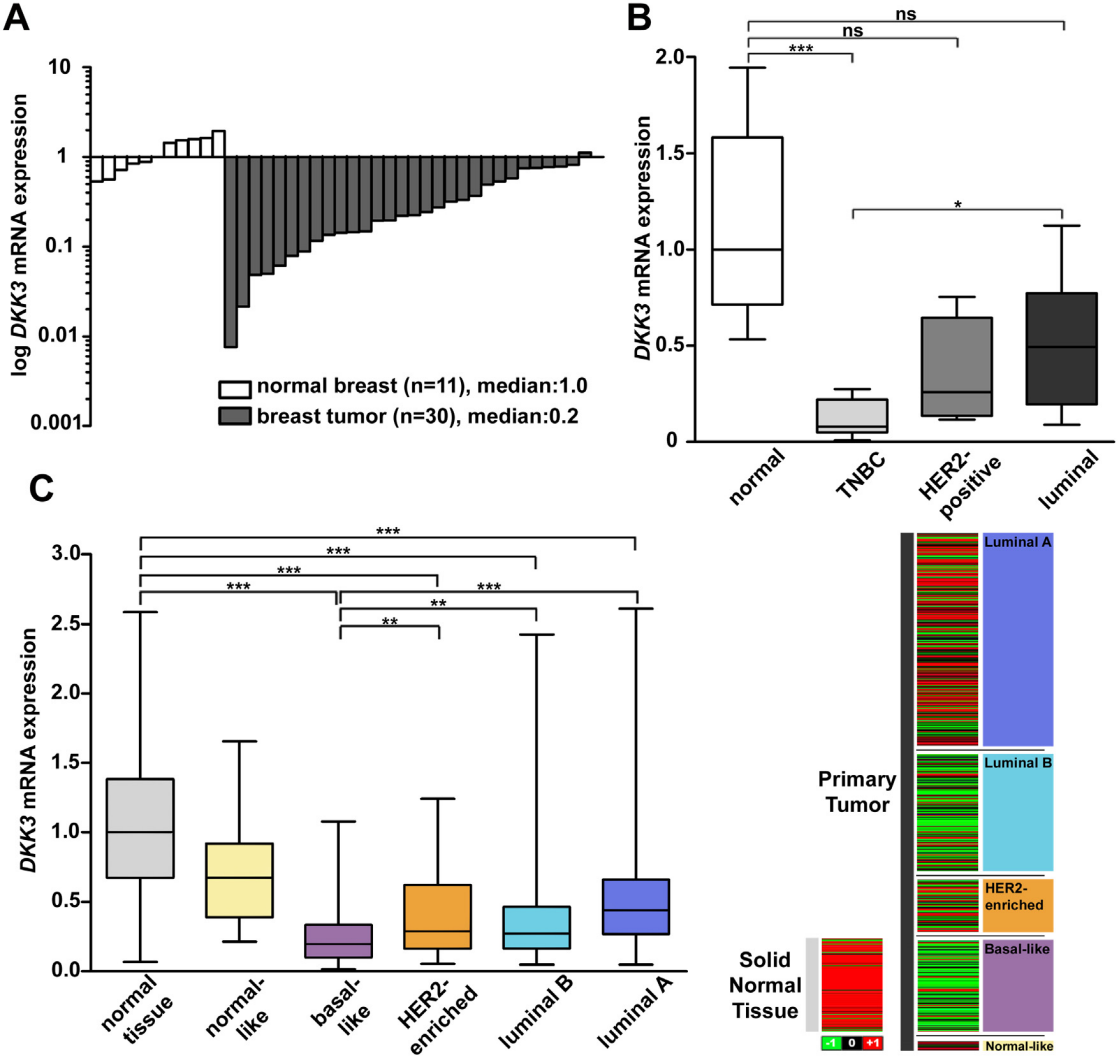
Down-regulation of *DKK3* mRNA expression in human breast cancer. (A) Real-time PCR-based *DKK3* mRNA expression analysis of 30 breast tumor compared to 11 healthy breast tissue samples. (B) Box plot of the samples shown in A demonstrating a down-regulation of *DKK3* mRNA in breast tumor compared to healthy breast tissue samples, especially in carcinomas of the IHC-defined triple negative breast cancer subtype (TNBC). (C) *In silico DKK3* mRNA expression analysis of 791 PAM50-defined breast tumor and 113 normal breast tissue samples depicted as heat map (left) and box plot (right). Red color: high, black: intermediate, green: low *DKK3* mRNA expression. Horizontal lines: grouped medians. Boxes: 25-75% quartiles. Vertical lines: range, minimum and maximum. ns: not significant, * *P* < 0.05, ** *P* < 0.01, *** *P* < 0.001.

In an earlier study of our group, we have already shown that *DKK3* expression is lost in human breast cancer by promoter-hypermethylation [36]. However, a subtype-specific expression analysis for DKK3 in breast cancer has not been performed so far. Using semi-quantitative real-time PCR, the *DKK3* mRNA expression in 30 subtype-stratified breast cancer and 11 healthy breast tissue samples was analyzed. Breast cancer tissues showed a median *DKK3* expression loss of 78% compared to healthy breast tissues (Fig 1A). Interestingly, we observed pronounced decrease in *DKK3* mRNA expression in the aggressive TNBC subtype. Compared to the expression in normal breast tissue samples, TNBC cases revealed a significantly reduced *DKK3* expression (median fold change (FC): 12.7, *P* < 0.001), while expression loss was less abundant in carcinomas of the HER2-positive (median FC: 3.9) and luminal subtype (median FC: 2, Fig 1B).

These findings are supported by a positive correlation of reduced *DKK3* expression with negative hormone receptor status (ER: *P* < 0.01 and PR: *P* = 0.066) as shown in the supplements (S6 Table). Furthermore low *DKK3* expression is significantly related to high tumor grade (*P* < 0.05, S6 Table), indicating a possible involvement of DKK3 in epithelial differentiation [37].

To evaluate the significance of our data, we analyzed *DKK3* mRNA levels in a large data set of an independent study. Using Illumina HiSeq expression data of 791 PAM50-defined breast cancer cases and 113 normal breast tissues available at TCGA portal [32], we verified a highly significant loss of *DKK3* mRNA expression in all clinically relevant breast cancer subtypes compared with normal breast tissues (for all *P* < 0.001). Supporting our data, strongest reduction was observed in the basal-like subtype (median FC: 5.1, Fig 1C).

Additionally, analysis of clinico-pathological parameters in the TCGA cohort revealed a significantly positive correlation between low *DKK3* expression and a negative hormone receptor (ER and PR, both *P* < 0.001) as well as a negative HER2 status (*P* < 0.001) and greater tumor size (*P* < 0.01, S7 Table), underlining an association between loss of DKK3 expression and the aggressive TNBC subgroup [3].

**Fig 2:**
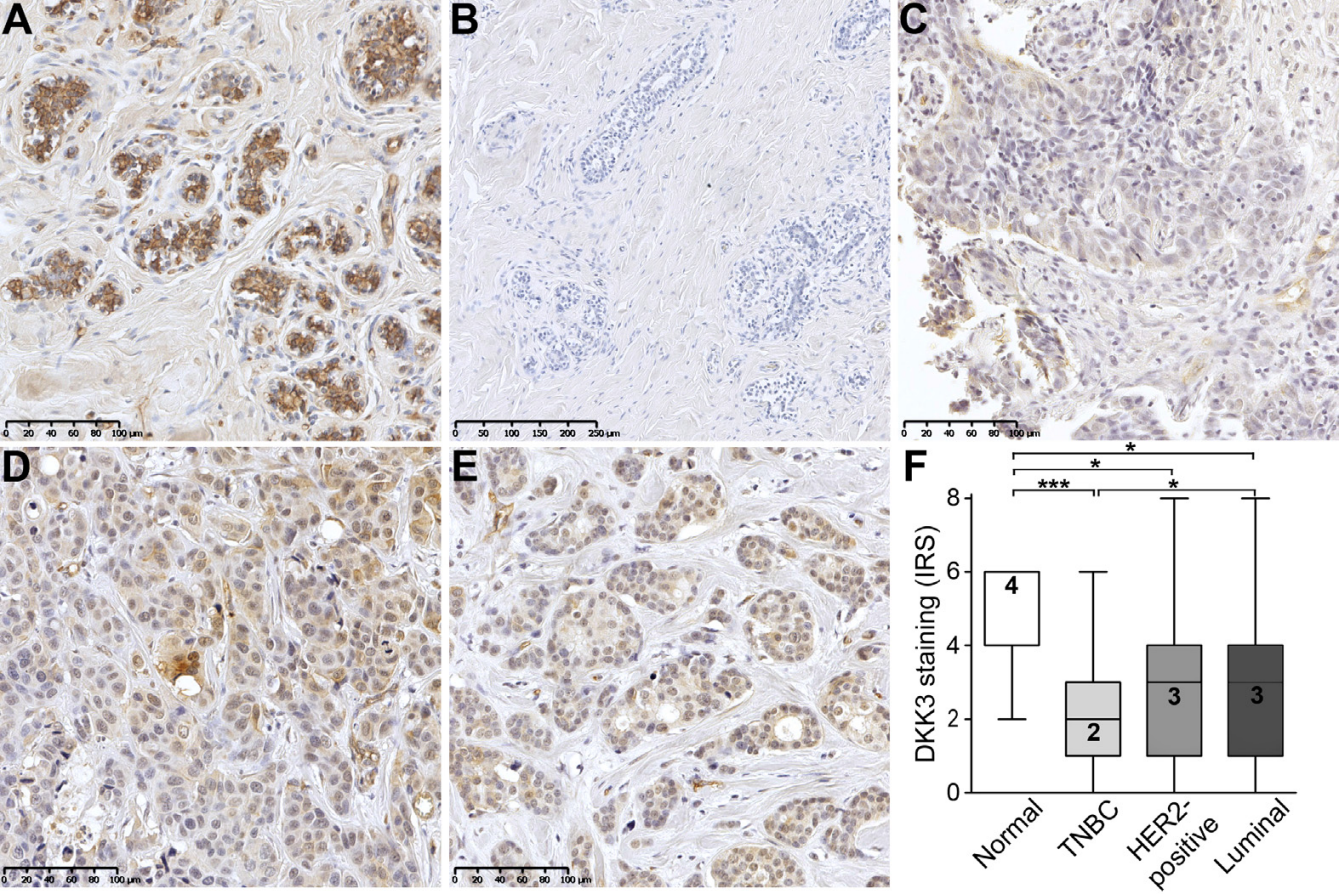
Loss of DKK3 protein expression in human breast cancer. (A) Normal mammary epithelial cells showing moderate, predominantly cytoplasmic, DKK3 immunoreactivity whereas (B) primary antibody negative control is free of signal. (C-E) Weak DKK3 protein expression is observed in breast tumor samples, with lowest intensity in IHC-defined (C) TNBC cases compared to (D) HER2-positive and (E) luminal carcinomas (representative images). (F) Box plot analysis demonstrating a significant down-regulation of DKK3 in tumors of the TNBC (n = 54), the HER2-positive (n = 47) and luminal subtype (n = 362) compared to normal breast tissues (n = 11). Horizontal lines: grouped medians. Boxes: 25-75% quartiles. Vertical lines: range, minimum and maximum. * *P* < 0.05, *** *P* < 0.001, IRS: immunoreactive score.

Next, we aimed to verify loss of DKK3 expression on protein level by performing immunohistochemistry analysis in normal (n = 11) and tumor (n = 463) breast tissues. DKK3 protein staining was quantified according to an adapted IRS [31]. In contrast to moderate, predominantly cytoplasmic DKK3 protein expression in the normal breast epithelium (median IRS = 4, Fig 2A), only weak DKK3 protein staining was observed in epithelial breast cancer cells (Fig 2C–2E). In concordance with the mRNA data, most abundant loss of DKK3 protein compared to normal breast tissue was noticed in the IHC-defined TNBC tumors (n = 54, median IRS = 2, *P* < 0.001, Fig 2C). Though, DKK3 protein expression was also significantly reduced in tumors of the HER2-positive (n = 47, median IRS = 3, *P* < 0.05, Fig 2D) and luminal subtype (n = 362, median IRS = 3, *P* < 0.05, Fig 2E).

### *DKK3* loss in human breast cancer is associated with an unfavorable patient prognosis

**Fig 3:**
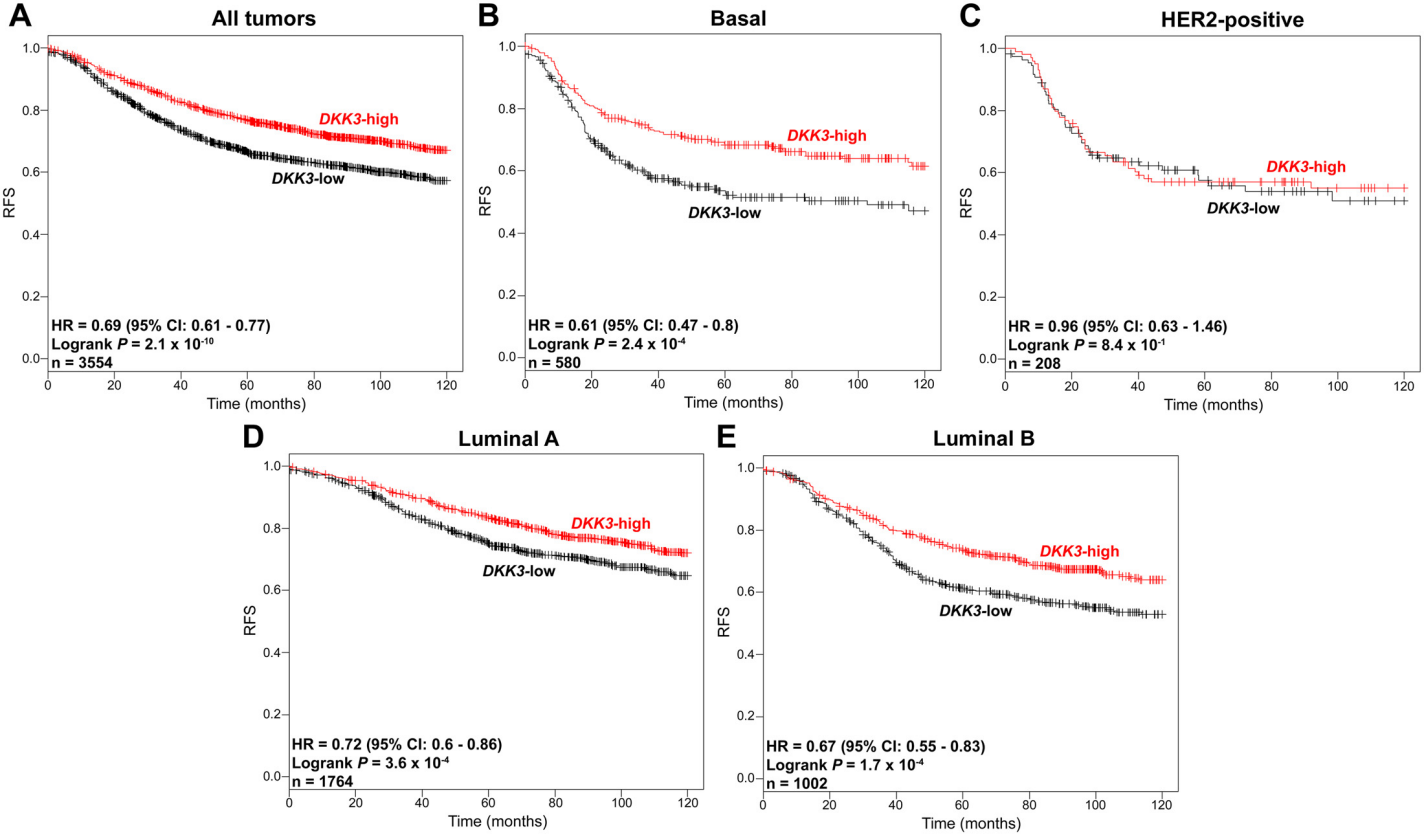
Loss of *DKK3* mRNA expression is associated with reduced recurrence-free survival (RFS) in breast cancer patients. Univariate Kaplan-Meier survival analysis of data obtained from the Kaplan-Meier Plotter portal illustrating RFS in patients with high *DKK3* (red curve) compared to patients with low *DKK3* mRNA expression (black curve) in (A) tumors of all subtypes, (B) basal subtype, (C) HER2-positive subtype, (D) luminal A subtype and (E) luminal B subtype. Vertical lines: censored cases. HR = Hazard ratio, 95% CI = 95% confidence interval.

Regarding the loss of DKK3 expression in tumor tissues, especially in basal cases demonstrated above, we next aimed at analyzing the clinical impact of *DKK3* expression on patient survival. In an earlier study of our group we have already shown that *DKK3* promoter-hypermethylation is associated with a poor prognosis (overall and disease free survival) in breast cancer patients [38]. However, a direct correlation of DKK3 expression and patient survival in the different molecular subtypes of breast cancer has not been analyzed so far. For this purpose univariate Kaplan-Meier survival analysis was performed, making use of a large dataset of the *Kaplan-Meier-Plotter* portal consisting of 3,554 breast cancer cases [34]. We noted that reduced *DKK3* mRNA expression was significantly associated (*P* < 0.001) with a shorter recurrence-free-survival of breast cancer patients (RFS, hazard ratio (HR): 0.69, Fig 3A).

Stratifying the breast cancer cohort further by subtype [39] revealed a potential prognostic relevance of *DKK3* mRNA expression in tumors of the basal (*P* < 0.001, Fig 3B) as well as the luminal subtype (*P* < 0.001, Fig 3D and 3E) but not in HER2-positive carcinomas (Fig 3C). Interestingly, strongest impact of DKK3 expression on patient survival was noticed in the group of basal cases (HR: 0.61) showing highest loss of DKK3 expression.

### DKK3 re-expression reduces cell growth possibly by inducing apoptosis in basal-like breast cancer cell lines

**Fig 4:**
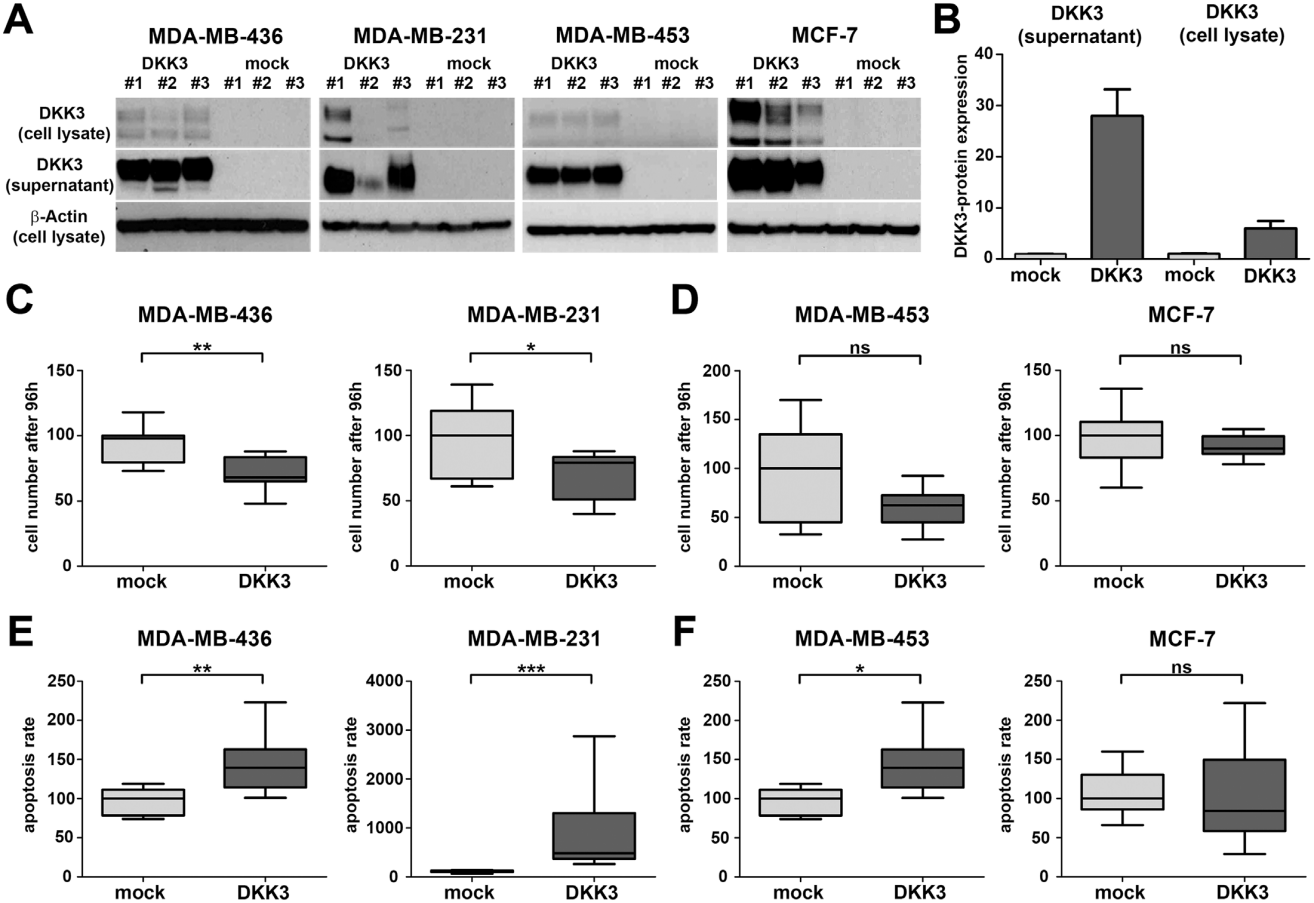
Stable DKK3 re-expression reduces cell growth of basal-like but not luminal-like breast cancer cell lines. (A) Re-expression of DKK3 protein as well as secretion of DKK3 was detected by western blot. Western blot analysis was performed on total cell lysates and corresponding cell culture supernatants of three stably transfected MDA-MB-436, MDA-MB-231, MDA-MB-453 and MCF-7 *DKK3* and mock clones respectively. β-actin served as a loading control. (B) All western blots depicted in A were evaluated densitometrically. In concordance, mock clones were negative for DKK3 protein whereas expression was elevated in total cell lysates of *DKK3* clones. Moreover, a strong secretion of DKK3 into the cell culture supernatant could only be detected in *DKK3* clones. The identical clones were used for the following functional assays. (C-D) Re-expression of DKK3 significantly reduced cell growth in basal-like (C, MDA-MB-436 and MDA-MB-231) but not luminal-like breast cancer cell lines (D, MDA-MB-453 and MCF-7). Box plots demonstrate the median cell number after 96 h cell growth of triplicate experiments. Cell growth suppression was possibly mediated by a DKK3-induced apoptosis, which was much more pronounced in breast cancer cell lines of the basal (E) than of the luminal subtype (F). Box plots demonstrate the median apoptosis rate of triplicate experiments. Horizontal lines: grouped medians. Boxes: 25-75% quartiles. Vertical lines: range, minimum and maximum. ns: not significant, * *P* < 0.05, ** *P* < 0.01, *** *P* < 0.001.

The potential prognostic impact of *DKK3* expression indicates a possible functional involvement of *DKK3* in the carcinogenesis of the human breast, especially in that of basal and luminal carcinomas. To address this hypothesis, we restored *DKK3* expression in two basal-like (MDA-MB-436 and MDA-MB-231) and two luminal-like breast cancer cell lines (MDA-MB-453 and MCF-7) by stable transfection with a full-length *DKK3* cDNA in a pcDNA3.1/V5-His-TOPO expression vector (*DKK3* clones) or an empty vector (mock clones). Ectopic DKK3 expression in stable *DKK3* clones was confirmed by real-time PCR (data not shown) and western blotting. As DKK3 is a secreted protein, DKK3 secretion into the cell culture supernatant was also checked by western blotting (Fig 4A and 4B).

Subsequently, we used these *in vitro* tumor models to analyze the effect of DKK3 re-expression on tumor cell behavior. Cell growth was determined by measurement of the cell number increase over a time period of 96 h. Ectopic expression of DKK3 significantly suppressed cell growth solely in basal-like but not in luminal-like breast cancer cell lines (MDA-MB-436, *P* < 0.01 and MDA-MB-231, *P* < 0.05, Fig 4C and 4D).

Next we evaluated if reduced cell growth in *DKK3* clones could be the result of enhanced induction of apoptosis. For this reason, we measured the activity of the effector caspases 3 and 7. We found significant increase in the activity of these caspases in three of the breast cancer cell lines analyzed (MDA-MB-436 *P* < 0.01, MDA-MB-231 *P* < 0.001, MDA-MB-453 *P* < 0.05). Again effects were most prominent in the two basal-like tumor models (compare Fig 4E and 4F). These findings suggest a possible involvement of DKK3 in the regulation of tumor cell growth, especially in the clinically highly relevant basal subtype.

### Re-expression of DKK3 might contribute to restoration of an epithelial phenotype in basal-like breast cancer cell lines

**Fig 5:**
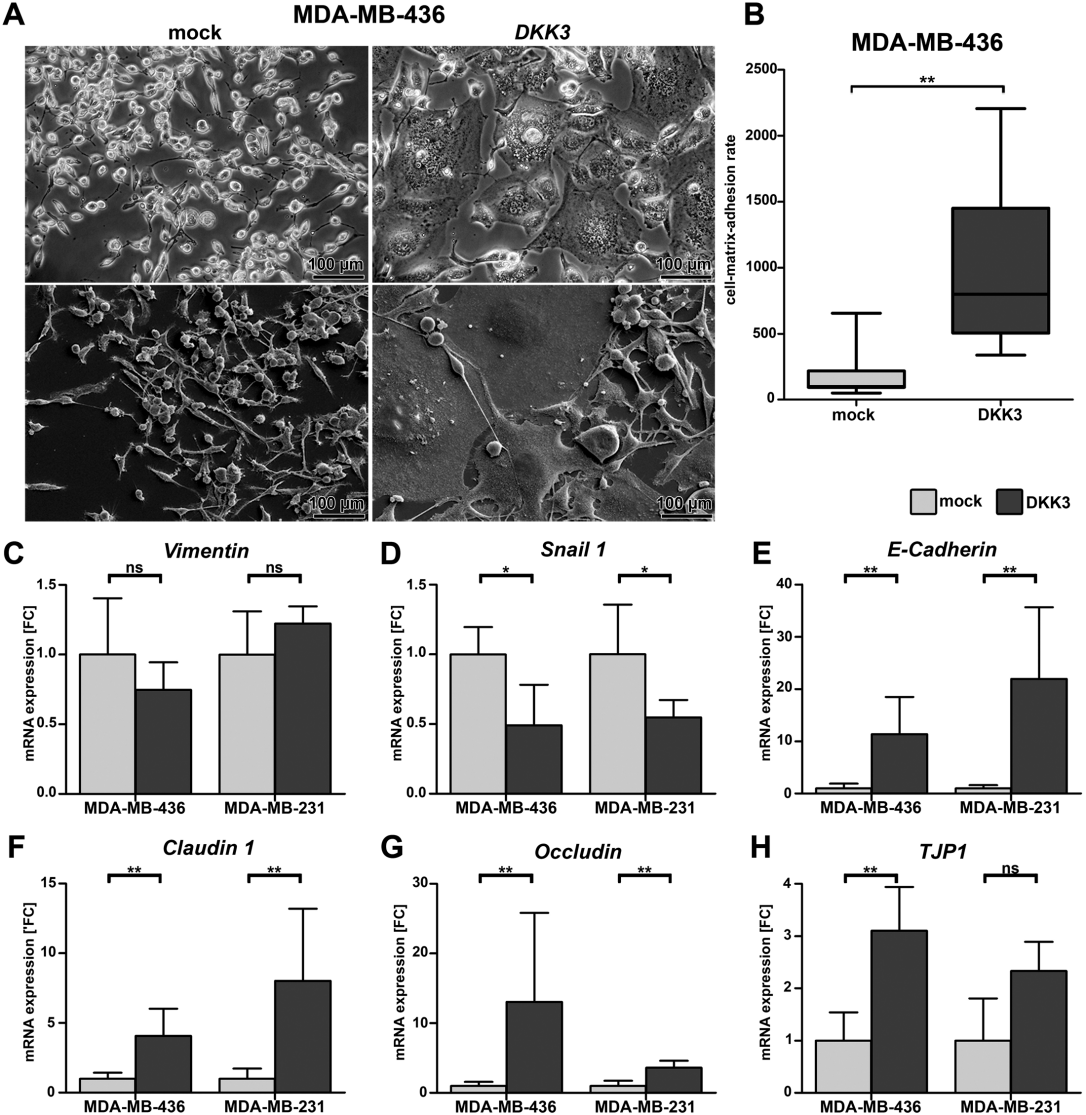
Ectopic expression of DKK3 leads to a modified cell morphology, enhanced cell-matrix-adhesion and increased expression of epithelial markers in basal-like breast cancer cells. (A) Altered cell morphology after re-expression of DKK3 in MDA-MB-436 cells displayed by phase-contrast microscopy (top) and scanning electron microscopy (bottom). Cells of the *DKK3* clones were enlarged and grew in clusters. (B) Change in morphology resulted in enhanced cell-matrix-adhesion of MDA-MB-436 *DKK3* clones (n = 3) on *Matrigel* compared to mock clones (n = 3). Experiments were performed in triplicate. Boxes: 25-75% quartiles. Vertical lines: range, minimum and maximum. Repressed mRNA expression of mesenchymal (C-D) and increased mRNA expression of epithelial markers (E-H) was revealed in *DKK3* clones (dark grey) compared to the corresponding mock clones (light grey) of the basal-like cell lines MDA-MB-436 and MDA-MB-231. Values represent the SD for 5 independent *DKK3* and mock clones each. FC: fold change, ns: not significant, * *P* < 0.05, ** *P* < 0.01.

Interestingly, DKK3 re-expression in mesenchymal-like MDA-MB-436 breast cancer cells resulted in a modified cell morphology. *DKK3* clones were characterized by a more epithelial-like appearance as they were enlarged and grew in cell clusters whereas mock clones were irregular shaped and displayed fibroblastic-like features (Fig 5A). Because changes in cell morphology are often accompanied by modifications in adhesive cell properties we next analyzed the cell adhesion to *Matrigel* which resembles the complex basement membrane matrix. As expected *DKK*3 clones of MDA-MB-436 cells adhered significantly faster to *Matrigel* compared to the mock clones indicating an enhanced cell-matrix adhesion (*P* < 0.01, Fig 5B). In contrast *DKK3* clones of basal-like MDA-MB-231 cells showed no change in cell morphology and cell-matrix adhesion rate (data not shown).

To reveal possible molecular alterations that might underlie the observed changes in cell morphology of MDA-MB-436 cells, we investigated the mRNA expression of well-known epithelial as well as mesenchymal markers in the two basal-like cell lines used in this study (Fig 5C–5H). No significant difference in expression of *Vimentin*, a major cytoskeletal component in mesenchymal cells, could be observed in both basal-like cell lines after DKK3 re-expression (MDA-MB-436 cells *P* = 0.42, MDA-MB-231 *P* = 0.31, Fig 5C). However, significant expression loss of the transcription factor *Snail 1* was revealed in *DKK3* clones of both cell lines (*P* < 0.01, Fig 5D) in line with an increased expression of the epithelial marker *E-Cadherin* (*P* < 0.05, Fig 5E) as Snail 1 promotes the repression of this gene [40, 41]. In addition, increased expression of epithelial markers known to be involved in the formation of cell-cell contacts like *Claudin 1* (both *P* < 0.01, Fig 5F), *Occludin* (both *P* < 0.01, Fig 5G) and *TJP1* (*Tight Junction Protein*, MDA-MB-436 cells *P* < 0.05, MDA-MB-231 *P* = 0.06, Fig 5H), could be shown for both basal-like cell lines after re-expression of DKK3. These results may indicate that loss of DKK3 expression during carcinogenesis of human breast cancer might contribute to the development of the aggressive mesenchymal phenotype in basal-type breast cancer.

## Discussion

It is appreciated that breast cancer is not simply one homogeneous disease but rather comprises multiple disease types with varying outcomes and responses to therapy. Breast carcinomas of the basal-like subtype have the poorest outcome together with luminal B and HER2-enriched tumors [4, 7]. But in contrast to luminal and HER2-enriched cancers, treatment options for basal-like tumors are still limited except for undirected chemotherapy [3, 8]. Therefore, novel targeted and less toxic therapies for this subtype are required. To achieve this goal, further understanding of molecular alterations underlying subtype-specific breast carcinogenesis is urgently need.

Loss of DKK3 has already been shown to be a consistent and widespread alteration among various human cancer types [21]. Likewise, decrease of DKK3 expression in human breast cancer was described before [36]. Here, we could confirm DKK3 expression loss in breast cancer specimens both on the mRNA and protein level. However, as breast cancer is a heterogeneous disease one goal of this study was to perform a comprehensive subtype-specific DKK3 expression analyses in human breast cancer for the first time. Interestingly the most abundant reduction of DKK3 expression was found in TNBC cases. This finding was supported by analysis of a large independent *DKK3* mRNA expression data set of breast cancer samples obtained from the TCGA platform [32]. Likewise, TCGA data revealed the lowest expression in the PAM50-defined basal subtype, which has a great overlap with IHC-defined TNBC [42]. Further support was obtained by the positive correlation between low *DKK3* mRNA expression and negative hormone receptor status using our own as well as the TCGA breast cancer data set. In addition pronounced expression loss of *DKK3* correlated with high tumor grade and greater tumor size in line with a high percentage of basal-like tumors exhibiting these characteristics [3].

Regarding the association between low *DKK3* expression and unfavorable tumor characteristics like high tumor grade and larger tumor size, we next aimed at analyzing the clinical impact of *DKK3* expression on patient survival. In the past our group already demonstrated an association between *DKK3* promoter-hypermethylation and poor patient survival in breast cancer patients [38], which was confirmed by Xiang *et al*. in an Asian population cohort [25]. However, the current study is the first to analyze the impact of *DKK3* mRNA expression on breast cancer patient survival. For this purpose, we made use of Affymetrix microarray expression and corresponding survival data obtained from the *Kaplan-Meier Plotter* portal to analyze the RFS in relation to *DKK3* mRNA expression. Univariate analysis demonstrated that patients with low *DKK3* expression had a highly significantly reduced RFS compared to those with exhibiting high *DKK3* expression. In the next step, we further stratified the breast cancer cohort into the molecular subtypes luminal A and B, HER2-positive and basal. Loss of *DKK3* expression served as a prognostic factor for unfavorable outcome in carcinomas of all subtypes except for HER2-postive cases. Nevertheless, strongest impact of *DKK3* expression on patient survival was noticed in the group of basal cases also showing highest loss of *DKK3* expression.

The potential prognostic impact of *DKK3* expression indicated a possible functional involvement of *DKK3* in the carcinogenesis of the human breast, especially in that of basal and luminal carcinomas. Previously, other studies could already demonstrate that DKK3 is able to suppress cell growth and to induce apoptosis in human breast cancer cell lines, indicating a tumor suppressive function for DKK3 [24, 25]. None of these reports focused on investigations of a possible subtype-specific function of DKK3 in the tumorigenesis of breast cancer. But a divergent DKK3 expression in the distinct breast cancer subtypes as well as a potential clinical impact for DKK3 only in basal and luminal tumors suggested a potential subtype-specific function of DKK3 in the development of human breast cancer. Therefore, we generated stable gain-of-function *in vitro* tumor models using two basal-like (MDA-MB-436 and MDA-MB-231) as well as two luminal-like (MDA-MB-453 and MCF-7) breast cancer cell lines. DKK3 re-expression resulted in cell growth suppression possibly mediated by up-regulation of apoptosis in the basal-like but not in the luminal-like breast cancer models. This indicates a role for DKK3 in tumor growth regulation most notably in basal breast cancers.

Epithelial-to-mesenchymal-transition (EMT) is defined by the loss of epithelial and gain of mesenchymal characteristics [43]. It is associated with tumor progression as well as invasion and in breast tumors EMT is mostly related to the aggressive basal subtype [44]. An association between loss of DKK3 expression and EMT in breast cancer has already been described by other groups [25, 26]. Xiang and colleagues observed a DKK3 mediated alteration of cell morphology and suggested, supported by expression data of well-known EMT-markers, an involvement of DKK3 in inhibiting EMT [25]. In the current study, we also noticed a modified cell morphology accompanied by a higher cell-matrix adhesion rate in one of our basal tumor models (MDA-MB-436) after stable re-expression of DKK3. In support of this observation, we noticed enhanced mRNA expression of the epithelial junction proteins E-Cadherin, Claudin 1, Occludin and TJP1 in independent stably re-expressing *DKK3* clones of both basal-like cell lines used in this study. Furthermore, the expression of Snail 1, a key EMT regulator and repressor of the E-Cadherin promoter [43, 45], was significantly reduced in *DKK3* clones compared to the corresponding mock clones. Though, no altered expression of Vimentin, a classic mesenchymal marker, could be shown. All in all, our data suggest that DKK3 clones exhibit attributes of both epithelial and mesenchymal phenotypes. Such a state was defined by Savagner as a “metastable phenotype” [43, 46], representing a partial EMT. But to further prove this hypothesis studies addressing cell motility and analysis of signaling pathways involved in the process of EMT will be necessary. Nevertheless, our findings suggest that DKK3 re-expression might contribute to restoration of an epithelial phenotype.

In conclusion, our findings provide for the first time evidence that DKK3 might have a subtype-specific function in human breast cancer. Loss of DKK3 expression is particularly observed in basal breast cancer and seems to be involved in the tumorigenesis of this subtype. *In vitro* studies reveal the impact of DKK3 on tumor growth, probably mediated by regulation of apoptosis, and on cell morphology modifications accompanied by changes on the molecular level. Hence, further studies analyzing the molecular mechanisms of these effects may help to identify novel targeted therapies for the clinically highly relevant basal breast cancer subtype.

## Supporting Information

S1 Table Cohort characteristics of cryoconserved breast cancer specimens. This table summarizes the clinico-pathological parameters of the fresh frozen breast cancer samples used in this study for RNA expression analyses.
(PDF)

S2 Table Cohort characteristics of FFPE breast cancer specimens. This table summarizes the clinico-pathological parameters of the FFPE breast cancer samples used in this study for protein expression analyses.
(PDF)

S3 Table TCGA breast cancer sample IDs. This table lists all TCGA breast cancer samples used for the *in silico* expression analysis of *DKK3* in this study.
(PDF)

S4 Table Cohort characteristics of TCGA *in silico* breast cancer specimens. This table summarizes the clinico-pathological parameters of the TCGA *in silico* breast cancer samples used in this study for mRNA expression analyses.
(PDF)

S5 Table Primer sequences and rt-PCR reaction conditions for expression analysis. This table illustrates the sequences of all primers used in this study, the rt-PCR reaction conditions and the size of the respective amplicon.
(PDF)

S6 Table Clinico-pathological parameters of cryoconserved breast cancer specimens in relation to DKK3 mRNA expression. This table summarizes the correlation between clinico-pathological parameters of cryoconserved breast cancer samples used in this study and corresponding DKK3 mRNA expression data.
(PDF)

S7 Table Clinico-pathological parameters of TCGA *in silico* breast cancer specimens in relation to DKK3 mRNA expression. This table summarizes the correlation between clinico-pathological parameters of TCGA *in silico* breast cancer samples used in this study and corresponding DKK3 mRNA expression data.
(PDF)
