# Supplementary material for: Loss of Dickkopf 3 Promotes the Tumorigenesis of Basal Breast Cancer
Source: PLoS One. 2016 Jul 28;11(7):e0160077. doi: 10.1371/journal.pone.0160077 (PMC4965070; doi:10.1371/journal.pone.0160077)
Supplement: S1 Table — This table summarizes the clinico-pathological parameters of the fresh frozen breast cancer samples used in this study for RNA expression analyses. (PDF) [file pone.0160077.s001.pdf]

Supplementary Table 1: Clinico-pathological parameters of 30 cryoconserved breast cancer specimens analyzed in this study.

Parameter	Categorization	n[a] analyzable	%
Age at diagnosis:	median 63 years (range 33-81)		
	<63 years	14	46.7
	≥63 years	16	53.3
Tumor size[b]	pT1	14	46.7
	pT2	15	50.0
	pT3	1	3.3
	pT4	0	0.0
Lymph node status[b]	pN0	17	56.7
	pN1-3	12	40.0
	unknown	1	3.3
Histological tumor grade[c]	G1	0	0.0
	G2	8	26.7
	G3	22	73.3
Oestrogen receptor status	negative (IRS[d] 0-2)	16	53.3
	positive (IRS[d] 3-12)	14	46.7
Progesterone receptor status	negative (IRS[d] 0-2)	15	50.0
	positive (IRS[d] 3-12)	15	50.0
HER2 status[e]	negative	24	80.0
	positive	6	20.0

[a]Only female patients with primary, unilateral, invasive breast cancer were included. [b]According to TNM classification by Sobin and Wittekind [1]. [c]According to Bloom and Richardson, as modified by Elston and Ellis [2]. [d]Immunoreactive score (IRS) according to Remmele and Stegner [3]. [e]Overexpression of the *ERBB2* gene (Her-2/neu) was diagnosed analogously to the threshold of the DAKO-Score system based on IHC assay. Uncertain cases were additionally validated by FISH assay. Percentages may not sum-up to 100% due to rounding.

1. Sobin LH, Fleming ID. TNM Classification of Malignant Tumors, fifth edition (1997). Union Internationale Contre le Cancer and the American Joint Committee on Cancer. Cancer. 1997;80(9):1803-4. PubMed PMID: 9351551;

2. Elston CW, Ellis IO. Pathological prognostic factors in breast cancer. I. The value of histological grade in breast cancer: experience from a large study with long-term follow-up. Histopathology. 1991;19(5):403-10. PubMed PMID: 1757079;

3. Remmele W, Stegner HE. Recommendation for uniform definition of an immunoreactive score (IRS) for immunohistochemical estrogen receptor detection (ER-ICA) in breast cancer tissue. Pathologe. 1987;8(3):138-40. PubMed PMID: 3303008;
