# Supplementary material for: Loss of Dickkopf 3 Promotes the Tumorigenesis of Basal Breast Cancer
Source: PLoS One. 2016 Jul 28;11(7):e0160077. doi: 10.1371/journal.pone.0160077 (PMC4965070; doi:10.1371/journal.pone.0160077)
Supplement: S2 Table — This table summarizes the clinico-pathological parameters of the FFPE breast cancer samples used in this study for protein expression analyses. (PDF) [file pone.0160077.s002.pdf]

Supplementary Table 2: Clinico-pathological parameters of 463 Formalin-fixed, paraffin-embedded (FFPE) breast cancer specimens analyzed in this study.

Parameter	Categorization	n[a] analyzable	%
Age at diagnosis:	median 51 years (range 21-66)		
	<51 years	192	41.5
	≥51 years	269	58.1
	unknown	2	0.4
Tumor size[b]	pT1	214	46.4
	pT2	219	47.5
	pT3	20	4.3
	pT4	5	1.1
	unknown	5	1.1
Lymph node status[b]	pN0	1	0.2
	pN1-3	457	99.1
	unknown	5	1.1
Histological tumor grade[c]	G1	24	5.2
	G2	274	59.4
	G3	154	33.4
	unknown	11	2.4
Oestrogen receptor status	negative (IRS[d] 0-2)	133	28.9
	positive (IRS[d] 3-12)	330	71.6
Progesterone receptor status	negative (IRS[d] 0-2)	161	34.9
	positive (IRS[d] 3-12)	302	65.5
HER2 status[e]	negative	323	69.8
	positive	140	30.2

[a]Only female patients with primary, unilateral, invasive breast cancer were included. [b]According to TNM classification by Sobin and Wittekind [1]. [c]According to Bloom and Richardson, as modified by Elston and Ellis [2]. [d]Immunoreactive score (IRS) according to Remmele and Stegner [3]. [e]Overexpression of the *ERBB2* gene (Her-2/neu) was diagnosed analogously to the threshold of the DAKO-Score system based on IHC assay. Uncertain cases were additionally validated by FISH assay. Percentages may not sum-up to 100% due to rounding.

1. Sobin LH, Fleming ID. TNM Classification of Malignant Tumors, fifth edition (1997). Union Internationale Contre le Cancer and the American Joint Committee on Cancer. Cancer. 1997;80(9):1803-4. PubMed PMID: 9351551;

2. Elston CW, Ellis IO. Pathological prognostic factors in breast cancer. I. The value of histological grade in breast cancer: experience from a large study with long-term follow-up. Histopathology. 1991;19(5):403-10. PubMed PMID: 1757079;

3. Remmele W, Stegner HE. Recommendation for uniform definition of an immunoreactive score (IRS) for immunohistochemical estrogen receptor detection (ER-ICA) in breast cancer tissue. Pathologe. 1987;8(3):138-40. PubMed PMID: 3303008;
