# Supplementary material for: Loss of Dickkopf 3 Promotes the Tumorigenesis of Basal Breast Cancer
Source: PLoS One. 2016 Jul 28;11(7):e0160077. doi: 10.1371/journal.pone.0160077 (PMC4965070; doi:10.1371/journal.pone.0160077)
Supplement: S3 Table — This table lists all TCGA breast cancer samples used for the in silico expression analysis of DKK3 in this study. (PDF) [file pone.0160077.s003.pdf]

TCGA breast cancer sample ID

TCGA-A1-A0SB-01
TCGA-A1-A0SD-01
TCGA-A1-A0SE-01
TCGA-A1-A0SF-01
TCGA-A1-A0SG-01
TCGA-A1-A0SH-01
TCGA-A1-A0SI-01
TCGA-A1-A0SJ-01
TCGA-A1-A0SK-01
TCGA-A1-A0SN-01
TCGA-A1-A0SO-01
TCGA-A1-A0SQ-01
TCGA-A2-A04N-01
TCGA-A2-A04P-01
TCGA-A2-A04Q-01
TCGA-A2-A04R-01
TCGA-A2-A04T-01
TCGA-A2-A04U-01
TCGA-A2-A04V-01
TCGA-A2-A04W-01
TCGA-A2-A04X-01
TCGA-A2-A04Y-01
TCGA-A2-A0CL-01
TCGA-A2-A0CM-01
TCGA-A2-A0CP-01
TCGA-A2-A0CQ-01
TCGA-A2-A0CS-01
TCGA-A2-A0CT-01
TCGA-A2-A0CU-01
TCGA-A2-A0CV-01
TCGA-A2-A0CW-01
TCGA-A2-A0CX-01
TCGA-A2-A0CZ-01
TCGA-A2-A0D0-01
TCGA-A2-A0D1-01
TCGA-A2-A0D2-01
TCGA-A2-A0D3-01
TCGA-A2-A0D4-01
TCGA-A2-A0EM-01
TCGA-A2-A0EN-01
TCGA-A2-A0EO-01
TCGA-A2-A0EQ-01
TCGA-A2-A0ER-01
TCGA-A2-A0ES-01
TCGA-A2-A0ET-01
TCGA-A2-A0EU-01
TCGA-A2-A0EV-01
TCGA-A2-A0EW-01

TCGA-A2-A0EX-01
TCGA-A2-A0EY-01
TCGA-A2-A0ST-01
TCGA-A2-A0SU-01
TCGA-A2-A0SV-01
TCGA-A2-A0SW-01
TCGA-A2-A0SX-01
TCGA-A2-A0SY-01
TCGA-A2-A0T0-01
TCGA-A2-A0T1-01
TCGA-A2-A0T2-01
TCGA-A2-A0T3-01
TCGA-A2-A0T4-01
TCGA-A2-A0T5-01
TCGA-A2-A0T6-01
TCGA-A2-A0T7-01
TCGA-A2-A0YC-01
TCGA-A2-A0YD-01
TCGA-A2-A0YE-01
TCGA-A2-A0YF-01
TCGA-A2-A0YG-01
TCGA-A2-A0YH-01
TCGA-A2-A0YI-01
TCGA-A2-A0YJ-01
TCGA-A2-A0YK-01
TCGA-A2-A0YL-01
TCGA-A2-A0YM-01
TCGA-A2-A1FV-01
TCGA-A2-A1FW-01
TCGA-A2-A1FX-01
TCGA-A2-A1FZ-01
TCGA-A2-A1G0-01
TCGA-A2-A1G1-01
TCGA-A2-A1G4-01
TCGA-A2-A1G6-01
TCGA-A2-A259-01
TCGA-A2-A25B-01
TCGA-A2-A25C-01
TCGA-A2-A25D-01
TCGA-A2-A25E-01
TCGA-A2-A25F-01
TCGA-A2-A3KC-01
TCGA-A2-A3KD-01
TCGA-A7-A0CE-01
TCGA-A7-A0CG-01
TCGA-A7-A0CH-01
TCGA-A7-A0CJ-01
TCGA-A7-A0D9-01
TCGA-A7-A0DA-01
TCGA-A7-A0DB-01

TCGA-A7-A13D-01
TCGA-A7-A13E-01
TCGA-A7-A13F-01
TCGA-A7-A26G-01
TCGA-A7-A26J-01
TCGA-A7-A3IY-01
TCGA-A7-A3IZ-01
TCGA-A7-A3J0-01
TCGA-A8-A06N-01
TCGA-A8-A06O-01
TCGA-A8-A06P-01
TCGA-A8-A06Q-01
TCGA-A8-A06R-01
TCGA-A8-A06X-01
TCGA-A8-A06Y-01
TCGA-A8-A06Z-01
TCGA-A8-A075-01
TCGA-A8-A076-01
TCGA-A8-A079-01
TCGA-A8-A07B-01
TCGA-A8-A07C-01
TCGA-A8-A07F-01
TCGA-A8-A07G-01
TCGA-A8-A07I-01
TCGA-A8-A07J-01
TCGA-A8-A07L-01
TCGA-A8-A07O-01
TCGA-A8-A07P-01
TCGA-A8-A07R-01
TCGA-A8-A07S-01
TCGA-A8-A07U-01
TCGA-A8-A07W-01
TCGA-A8-A081-01
TCGA-A8-A082-01
TCGA-A8-A083-01
TCGA-A8-A086-01
TCGA-A8-A08B-01
TCGA-A8-A08C-01
TCGA-A8-A08G-01
TCGA-A8-A08H-01
TCGA-A8-A08I-01
TCGA-A8-A08J-01
TCGA-A8-A08L-01
TCGA-A8-A08O-01
TCGA-A8-A08P-01
TCGA-A8-A08R-01
TCGA-A8-A08T-01
TCGA-A8-A08X-01
TCGA-A8-A08Z-01
TCGA-A8-A090-01

TCGA-A8-A092-01
TCGA-A8-A094-01
TCGA-A8-A095-01
TCGA-A8-A096-01
TCGA-A8-A097-01
TCGA-A8-A099-01
TCGA-A8-A09A-01
TCGA-A8-A09B-01
TCGA-A8-A09D-01
TCGA-A8-A09G-01
TCGA-A8-A09M-01
TCGA-A8-A09N-01
TCGA-A8-A09Q-01
TCGA-A8-A09R-01
TCGA-A8-A09T-01
TCGA-A8-A09V-01
TCGA-A8-A09W-01
TCGA-A8-A09X-01
TCGA-A8-A0A1-01
TCGA-A8-A0A2-01
TCGA-A8-A0A4-01
TCGA-A8-A0A6-01
TCGA-A8-A0A7-01
TCGA-A8-A0A9-01
TCGA-A8-A0AB-01
TCGA-A8-A0AD-01
TCGA-AC-A23C-01
TCGA-AC-A23E-01
TCGA-AC-A23G-01
TCGA-AC-A23H-01
TCGA-AC-A2B8-01
TCGA-AC-A2BK-01
TCGA-AC-A2BM-01
TCGA-AC-A2FB-01
TCGA-AC-A2FF-01
TCGA-AC-A2FG-01
TCGA-AC-A2FK-01
TCGA-AC-A2FO-01
TCGA-AC-A2QH-01
TCGA-AC-A3HN-01
TCGA-AC-A3OD-01
TCGA-AN-A03X-01
TCGA-AN-A03Y-01
TCGA-AN-A041-01
TCGA-AN-A046-01
TCGA-AN-A049-01
TCGA-AN-A04A-01
TCGA-AN-A04C-01
TCGA-AN-A04D-01
TCGA-AN-A0AJ-01

TCGA-AN-A0AK-01
TCGA-AN-A0AL-01
TCGA-AN-A0AM-01
TCGA-AN-A0AS-01
TCGA-AN-A0AT-01
TCGA-AN-A0FD-01
TCGA-AN-A0FF-01
TCGA-AN-A0FJ-01
TCGA-AN-A0FK-01
TCGA-AN-A0FL-01
TCGA-AN-A0FN-01
TCGA-AN-A0FS-01
TCGA-AN-A0FT-01
TCGA-AN-A0FV-01
TCGA-AN-A0FW-01
TCGA-AN-A0FX-01
TCGA-AN-A0FY-01
TCGA-AN-A0FZ-01
TCGA-AN-A0G0-01
TCGA-AN-A0XL-01
TCGA-AN-A0XN-01
TCGA-AN-A0XO-01
TCGA-AN-A0XP-01
TCGA-AN-A0XR-01
TCGA-AN-A0XS-01
TCGA-AN-A0XT-01
TCGA-AN-A0XU-01
TCGA-AN-A0XV-01
TCGA-AN-A0XW-01
TCGA-AO-A03L-01
TCGA-AO-A03M-01
TCGA-AO-A03N-01
TCGA-AO-A03O-01
TCGA-AO-A03P-01
TCGA-AO-A03R-01
TCGA-AO-A03T-01
TCGA-AO-A03U-01
TCGA-AO-A03V-01
TCGA-AO-A0J2-01
TCGA-AO-A0J3-01
TCGA-AO-A0J4-01
TCGA-AO-A0J6-01
TCGA-AO-A0J7-01
TCGA-AO-A0J8-01
TCGA-AO-A0J9-01
TCGA-AO-A0JA-01
TCGA-AO-A0JC-01
TCGA-AO-A0JD-01
TCGA-AO-A0JE-01
TCGA-AO-A0JF-01

TCGA-AO-A0JG-01
TCGA-AO-A0JI-01
TCGA-AO-A0JJ-01
TCGA-AO-A0JL-01
TCGA-AO-A0JM-01
TCGA-AO-A124-01
TCGA-AO-A125-01
TCGA-AO-A126-01
TCGA-AO-A128-01
TCGA-AO-A129-01
TCGA-AO-A12A-01
TCGA-AO-A12B-01
TCGA-AO-A12C-01
TCGA-AO-A12D-01
TCGA-AO-A12E-01
TCGA-AO-A12F-01
TCGA-AO-A12G-01
TCGA-AO-A12H-01
TCGA-AO-A1KO-01
TCGA-AO-A1KP-01
TCGA-AO-A1KR-01
TCGA-AO-A1KS-01
TCGA-AO-A1KT-01
TCGA-AQ-A04H-01
TCGA-AQ-A04J-01
TCGA-AQ-A04L-01
TCGA-AQ-A1H2-01
TCGA-AQ-A1H3-01
TCGA-AR-A0TP-01
TCGA-AR-A0TQ-01
TCGA-AR-A0TR-01
TCGA-AR-A0TS-01
TCGA-AR-A0TT-01
TCGA-AR-A0TU-01
TCGA-AR-A0TV-01
TCGA-AR-A0TW-01
TCGA-AR-A0TX-01
TCGA-AR-A0TY-01
TCGA-AR-A0TZ-01
TCGA-AR-A0U0-01
TCGA-AR-A0U2-01
TCGA-AR-A0U3-01
TCGA-AR-A0U4-01
TCGA-AR-A1AH-01
TCGA-AR-A1AI-01
TCGA-AR-A1AJ-01
TCGA-AR-A1AK-01
TCGA-AR-A1AL-01
TCGA-AR-A1AN-01
TCGA-AR-A1AO-01

TCGA-AR-A1AP-01
TCGA-AR-A1AQ-01
TCGA-AR-A1AS-01
TCGA-AR-A1AU-01
TCGA-AR-A1AW-01
TCGA-AR-A1AX-01
TCGA-AR-A1AY-01
TCGA-AR-A24H-01
TCGA-AR-A24K-01
TCGA-AR-A24L-01
TCGA-AR-A24M-01
TCGA-AR-A24N-01
TCGA-AR-A24O-01
TCGA-AR-A24P-01
TCGA-AR-A24Q-01
TCGA-AR-A24R-01
TCGA-AR-A24S-01
TCGA-AR-A24T-01
TCGA-AR-A24U-01
TCGA-AR-A24V-01
TCGA-AR-A24W-01
TCGA-AR-A24X-01
TCGA-AR-A24Z-01
TCGA-AR-A250-01
TCGA-AR-A251-01
TCGA-AR-A252-01
TCGA-AR-A254-01
TCGA-AR-A255-01
TCGA-AR-A256-01
TCGA-AR-A2LE-01
TCGA-AR-A2LH-01
TCGA-AR-A2LK-01
TCGA-AR-A2LM-01
TCGA-AR-A2LN-01
TCGA-AR-A2LO-01
TCGA-AR-A2LQ-01
TCGA-B6-A0I1-01
TCGA-B6-A0I2-01
TCGA-B6-A0I5-01
TCGA-B6-A0I6-01
TCGA-B6-A0I9-01
TCGA-B6-A0IA-01
TCGA-B6-A0IB-01
TCGA-B6-A0IC-01
TCGA-B6-A0IE-01
TCGA-B6-A0IG-01
TCGA-B6-A0IH-01
TCGA-B6-A0IJ-01
TCGA-B6-A0IK-01
TCGA-B6-A0IM-01

TCGA-B6-A0IN-01
TCGA-B6-A0IO-01
TCGA-B6-A0IP-01
TCGA-B6-A0IQ-01
TCGA-B6-A0RE-01
TCGA-B6-A0RG-01
TCGA-B6-A0RH-01
TCGA-B6-A0RI-01
TCGA-B6-A0RL-01
TCGA-B6-A0RM-01
TCGA-B6-A0RN-01
TCGA-B6-A0RO-01
TCGA-B6-A0RP-01
TCGA-B6-A0RQ-01
TCGA-B6-A0RS-01
TCGA-B6-A0RT-01
TCGA-B6-A0RU-01
TCGA-B6-A0RV-01
TCGA-B6-A0WS-01
TCGA-B6-A0WT-01
TCGA-B6-A0WV-01
TCGA-B6-A0WW-01
TCGA-B6-A0WX-01
TCGA-B6-A0WY-01
TCGA-B6-A0WZ-01
TCGA-B6-A0X0-01
TCGA-B6-A0X1-01
TCGA-B6-A0X4-01
TCGA-B6-A0X5-01
TCGA-B6-A0X7-01
TCGA-B6-A1KC-01
TCGA-B6-A1KF-01
TCGA-B6-A1KI-01
TCGA-B6-A1KN-01
TCGA-B6-A2IU-01
TCGA-BH-A0AU-01
TCGA-BH-A0AV-01
TCGA-BH-A0AW-01
TCGA-BH-A0AY-01
TCGA-BH-A0AZ-01
TCGA-BH-A0B0-01
TCGA-BH-A0B1-01
TCGA-BH-A0B3-01
TCGA-BH-A0B5-01
TCGA-BH-A0B7-01
TCGA-BH-A0B8-01
TCGA-BH-A0BA-01
TCGA-BH-A0BC-01
TCGA-BH-A0BD-01
TCGA-BH-A0BF-01

TCGA-BH-A0BG-01
TCGA-BH-A0BJ-01
TCGA-BH-A0BL-01
TCGA-BH-A0BM-01
TCGA-BH-A0BO-01
TCGA-BH-A0BP-01
TCGA-BH-A0BQ-01
TCGA-BH-A0BR-01
TCGA-BH-A0BS-01
TCGA-BH-A0BT-01
TCGA-BH-A0BV-01
TCGA-BH-A0BW-01
TCGA-BH-A0BZ-01
TCGA-BH-A0C0-01
TCGA-BH-A0C1-01
TCGA-BH-A0C3-01
TCGA-BH-A0C7-01
TCGA-BH-A0DE-01
TCGA-BH-A0DG-01
TCGA-BH-A0DH-01
TCGA-BH-A0DI-01
TCGA-BH-A0DK-01
TCGA-BH-A0DL-01
TCGA-BH-A0DP-01
TCGA-BH-A0DQ-01
TCGA-BH-A0DS-01
TCGA-BH-A0DT-01
TCGA-BH-A0DV-01
TCGA-BH-A0DX-01
TCGA-BH-A0DZ-01
TCGA-BH-A0E0-01
TCGA-BH-A0E1-01
TCGA-BH-A0E2-01
TCGA-BH-A0E6-01
TCGA-BH-A0E7-01
TCGA-BH-A0E9-01
TCGA-BH-A0EB-01
TCGA-BH-A0EE-01
TCGA-BH-A0EI-01
TCGA-BH-A0GY-01
TCGA-BH-A0GZ-01
TCGA-BH-A0H0-01
TCGA-BH-A0H3-01
TCGA-BH-A0H5-01
TCGA-BH-A0H6-01
TCGA-BH-A0H7-01
TCGA-BH-A0H9-01
TCGA-BH-A0HA-01
TCGA-BH-A0HB-01
TCGA-BH-A0HF-01

TCGA-BH-A0HI-01
TCGA-BH-A0HK-01
TCGA-BH-A0HO-01
TCGA-BH-A0HP-01
TCGA-BH-A0HQ-01
TCGA-BH-A0HU-01
TCGA-BH-A0HW-01
TCGA-BH-A0HX-01
TCGA-BH-A0HY-01
TCGA-BH-A0RX-01
TCGA-BH-A0W3-01
TCGA-BH-A0W4-01
TCGA-BH-A0W5-01
TCGA-BH-A0W7-01
TCGA-BH-A0WA-01
TCGA-BH-A18F-01
TCGA-BH-A18G-01
TCGA-BH-A18H-01
TCGA-BH-A18I-01
TCGA-BH-A18J-01
TCGA-BH-A18K-01
TCGA-BH-A18L-01
TCGA-BH-A18M-01
TCGA-BH-A18N-01
TCGA-BH-A18P-01
TCGA-BH-A18Q-01
TCGA-BH-A18R-01
TCGA-BH-A18S-01
TCGA-BH-A18T-01
TCGA-BH-A18U-01
TCGA-BH-A18V-01
TCGA-BH-A1EN-01
TCGA-BH-A1EO-01
TCGA-BH-A1ES-01
TCGA-BH-A1ET-01
TCGA-BH-A1EU-01
TCGA-BH-A1EV-01
TCGA-BH-A1EW-01
TCGA-BH-A1EX-01
TCGA-BH-A1EY-01
TCGA-BH-A1F0-01
TCGA-BH-A1F2-01
TCGA-BH-A1F6-01
TCGA-BH-A1F8-01
TCGA-BH-A1FB-01
TCGA-BH-A1FC-01
TCGA-BH-A1FD-01
TCGA-BH-A1FE-01
TCGA-BH-A1FG-01
TCGA-BH-A1FH-01

TCGA-BH-A1FJ-01
TCGA-BH-A1FL-01
TCGA-BH-A1FM-01
TCGA-BH-A1FN-01
TCGA-BH-A1FR-01
TCGA-BH-A1FU-01
TCGA-BH-A201-01
TCGA-BH-A202-01
TCGA-BH-A203-01
TCGA-BH-A204-01
TCGA-BH-A208-01
TCGA-BH-A209-01
TCGA-BH-A28Q-01
TCGA-BH-A2L8-01
TCGA-C8-A12K-01
TCGA-C8-A12L-01
TCGA-C8-A12M-01
TCGA-C8-A12N-01
TCGA-C8-A12O-01
TCGA-C8-A12P-01
TCGA-C8-A12Q-01
TCGA-C8-A12T-01
TCGA-C8-A12U-01
TCGA-C8-A12V-01
TCGA-C8-A12W-01
TCGA-C8-A12X-01
TCGA-C8-A12Y-01
TCGA-C8-A12Z-01
TCGA-C8-A130-01
TCGA-C8-A131-01
TCGA-C8-A132-01
TCGA-C8-A133-01
TCGA-C8-A134-01
TCGA-C8-A135-01
TCGA-C8-A137-01
TCGA-C8-A138-01
TCGA-C8-A1HE-01
TCGA-C8-A1HF-01
TCGA-C8-A1HG-01
TCGA-C8-A1HJ-01
TCGA-C8-A1HK-01
TCGA-C8-A1HM-01
TCGA-C8-A1HN-01
TCGA-C8-A1HO-01
TCGA-C8-A26V-01
TCGA-C8-A26W-01
TCGA-C8-A26X-01
TCGA-C8-A26Y-01
TCGA-C8-A26Z-01
TCGA-C8-A273-01

TCGA-C8-A274-01
TCGA-C8-A275-01
TCGA-C8-A278-01
TCGA-C8-A27A-01
TCGA-C8-A27B-01
TCGA-C8-A3M7-01
TCGA-C8-A3M8-01
TCGA-D8-A13Y-01
TCGA-D8-A13Z-01
TCGA-D8-A140-01
TCGA-D8-A141-01
TCGA-D8-A142-01
TCGA-D8-A143-01
TCGA-D8-A145-01
TCGA-D8-A147-01
TCGA-D8-A1J8-01
TCGA-D8-A1J9-01
TCGA-D8-A1JA-01
TCGA-D8-A1JB-01
TCGA-D8-A1JC-01
TCGA-D8-A1JD-01
TCGA-D8-A1JE-01
TCGA-D8-A1JF-01
TCGA-D8-A1JG-01
TCGA-D8-A1JH-01
TCGA-D8-A1JI-01
TCGA-D8-A1JJ-01
TCGA-D8-A1JK-01
TCGA-D8-A1JL-01
TCGA-D8-A1JM-01
TCGA-D8-A1JN-01
TCGA-D8-A1JP-01
TCGA-D8-A1JS-01
TCGA-D8-A1JT-01
TCGA-D8-A1JU-01
TCGA-D8-A1X5-01
TCGA-D8-A1X6-01
TCGA-D8-A1X7-01
TCGA-D8-A1X8-01
TCGA-D8-A1X9-01
TCGA-D8-A1XA-01
TCGA-D8-A1XB-01
TCGA-D8-A1XC-01
TCGA-D8-A1XD-01
TCGA-D8-A1XF-01
TCGA-D8-A1XG-01
TCGA-D8-A1XJ-01
TCGA-D8-A1XK-01
TCGA-D8-A1XL-01
TCGA-D8-A1XM-01

TCGA-D8-A1XO-01
TCGA-D8-A1XQ-01
TCGA-D8-A1XR-01
TCGA-D8-A1XT-01
TCGA-D8-A1XU-01
TCGA-D8-A1XV-01
TCGA-D8-A1XW-01
TCGA-D8-A1XY-01
TCGA-D8-A1XZ-01
TCGA-D8-A1Y0-01
TCGA-D8-A1Y1-01
TCGA-D8-A1Y2-01
TCGA-D8-A1Y3-01
TCGA-D8-A27E-01
TCGA-D8-A27F-01
TCGA-D8-A27G-01
TCGA-D8-A27H-01
TCGA-D8-A27I-01
TCGA-D8-A27K-01
TCGA-D8-A27L-01
TCGA-D8-A27M-01
TCGA-D8-A27N-01
TCGA-D8-A27P-01
TCGA-D8-A27R-01
TCGA-D8-A27T-01
TCGA-D8-A27V-01
TCGA-D8-A27W-01
TCGA-E2-A105-01
TCGA-E2-A106-01
TCGA-E2-A108-01
TCGA-E2-A109-01
TCGA-E2-A10A-01
TCGA-E2-A10B-01
TCGA-E2-A10C-01
TCGA-E2-A10E-01
TCGA-E2-A10F-01
TCGA-E2-A14N-01
TCGA-E2-A14O-01
TCGA-E2-A14P-01
TCGA-E2-A14Q-01
TCGA-E2-A14R-01
TCGA-E2-A14S-01
TCGA-E2-A14T-01
TCGA-E2-A14V-01
TCGA-E2-A14X-01
TCGA-E2-A14Y-01
TCGA-E2-A14Z-01
TCGA-E2-A150-01
TCGA-E2-A152-01
TCGA-E2-A153-01

TCGA-E2-A154-01
TCGA-E2-A155-01
TCGA-E2-A156-01
TCGA-E2-A158-01
TCGA-E2-A159-01
TCGA-E2-A15A-01
TCGA-E2-A15C-01
TCGA-E2-A15D-01
TCGA-E2-A15E-01
TCGA-E2-A15G-01
TCGA-E2-A15H-01
TCGA-E2-A15I-01
TCGA-E2-A15J-01
TCGA-E2-A15K-01
TCGA-E2-A15L-01
TCGA-E2-A15M-01
TCGA-E2-A15P-01
TCGA-E2-A15R-01
TCGA-E2-A15S-01
TCGA-E2-A1AZ-01
TCGA-E2-A1B0-01
TCGA-E2-A1B1-01
TCGA-E2-A1B4-01
TCGA-E2-A1B5-01
TCGA-E2-A1BD-01
TCGA-E2-A1IE-01
TCGA-E2-A1IF-01
TCGA-E2-A1IG-01
TCGA-E2-A1IH-01
TCGA-E2-A1II-01
TCGA-E2-A1IJ-01
TCGA-E2-A1IK-01
TCGA-E2-A1IL-01
TCGA-E2-A1IN-01
TCGA-E2-A1IO-01
TCGA-E2-A1IU-01
TCGA-E2-A1L6-01
TCGA-E2-A1L7-01
TCGA-E2-A1L9-01
TCGA-E2-A1LA-01
TCGA-E2-A1LB-01
TCGA-E2-A1LH-01
TCGA-E2-A1LI-01
TCGA-E2-A1LK-01
TCGA-E2-A1LL-01
TCGA-E2-A1LS-01
TCGA-E2-A3DX-01
TCGA-E9-A1N3-01
TCGA-E9-A1N4-01
TCGA-E9-A1N5-01

TCGA-E9-A1N6-01
TCGA-E9-A1N8-01
TCGA-E9-A1N9-01
TCGA-E9-A1NA-01
TCGA-E9-A1ND-01
TCGA-E9-A1NE-01
TCGA-E9-A1NF-01
TCGA-E9-A1NG-01
TCGA-E9-A1NH-01
TCGA-E9-A1NI-01
TCGA-E9-A1QZ-01
TCGA-E9-A1R0-01
TCGA-E9-A1R2-01
TCGA-E9-A1R3-01
TCGA-E9-A1R4-01
TCGA-E9-A1R5-01
TCGA-E9-A1R6-01
TCGA-E9-A1R7-01
TCGA-E9-A1RB-01
TCGA-E9-A1RC-01
TCGA-E9-A1RD-01
TCGA-E9-A1RE-01
TCGA-E9-A1RF-01
TCGA-E9-A1RH-01
TCGA-E9-A1RI-01
TCGA-E9-A226-01
TCGA-E9-A227-01
TCGA-E9-A228-01
TCGA-E9-A229-01
TCGA-E9-A22A-01
TCGA-E9-A22B-01
TCGA-E9-A22D-01
TCGA-E9-A22E-01
TCGA-E9-A22G-01
TCGA-E9-A22H-01
TCGA-E9-A243-01
TCGA-E9-A244-01
TCGA-E9-A245-01
TCGA-E9-A247-01
TCGA-E9-A248-01
TCGA-E9-A249-01
TCGA-E9-A24A-01
TCGA-E9-A295-01
TCGA-E9-A2JS-01
TCGA-E9-A2JT-01
TCGA-E9-A3HO-01
TCGA-E9-A3Q9-01
TCGA-EW-A1IW-01
TCGA-EW-A1IX-01
TCGA-EW-A1IY-01

TCGA-EW-A1J1-01
TCGA-EW-A1J2-01
TCGA-EW-A1J3-01
TCGA-EW-A1J5-01
TCGA-EW-A1J6-01
TCGA-EW-A1OV-01
TCGA-EW-A1OW-01
TCGA-EW-A1OX-01
TCGA-EW-A1OY-01
TCGA-EW-A1OZ-01
TCGA-EW-A1P0-01
TCGA-EW-A1P4-01
TCGA-EW-A1P5-01
TCGA-EW-A1P6-01
TCGA-EW-A1P7-01
TCGA-EW-A1P8-01
TCGA-EW-A1PA-01
TCGA-EW-A1PB-01
TCGA-EW-A1PC-01
TCGA-EW-A1PE-01
TCGA-EW-A1PF-01
TCGA-EW-A1PG-01
TCGA-EW-A1PH-01
TCGA-EW-A2FR-01
TCGA-EW-A2FS-01
TCGA-EW-A2FV-01
TCGA-EW-A2FW-01
TCGA-GI-A2C9-01
TCGA-GM-A2D9-01
TCGA-GM-A2DA-01
TCGA-GM-A2DB-01
TCGA-GM-A2DC-01
TCGA-GM-A2DD-01
TCGA-GM-A2DF-01
TCGA-GM-A2DH-01
TCGA-GM-A2DI-01
TCGA-GM-A2DK-01
TCGA-GM-A2DL-01
TCGA-GM-A2DM-01
TCGA-GM-A2DN-01
TCGA-GM-A2DO-01
TCGA-GM-A3NY-01
TCGA-HN-A2NL-01
