# Supplementary material for: Loss of Dickkopf 3 Promotes the Tumorigenesis of Basal Breast Cancer
Source: PLoS One. 2016 Jul 28;11(7):e0160077. doi: 10.1371/journal.pone.0160077 (PMC4965070; doi:10.1371/journal.pone.0160077)
Supplement: S4 Table — This table summarizes the clinico-pathological parameters of the TCGA in silico breast cancer samples used in this study for mRNA expression analyses. (PDF) [file pone.0160077.s004.pdf]

Supplementary Table 4: Clinico-pathological parameters of 791 breast cancer specimens obtained from *The Cancer Genome Atlas* platform.

Parameter	Categorisation	n[a] analysable	%
Age at diagnosis:	median 58 years (range 26-90)		
	≤58 years	409	51.7
	>58 years	382	48.3
Tumour size[b]	pT1	209	26.4
	pT2	468	59.2
	pT3	83	10.5
	pT4	28	3.5
	unknown	3	0.4
Lymph node status[b]	pN0	375	47.4
	pN1-3	403	51.0
	unknown	13	1.6
Oestrogen receptor status	negative (IRS[c] 0-2)	176	22.3
	positive (IRS[c] 3-12)	577	73.0
	unknown	38	4.8
Progesterone receptor status	negative (IRS[c] 0-2)	246	31.1
	positive (IRS[c] 3-12)	504	63.7
	unknown	41	5.2
HER2 status[d]	negative	600	75.9
	positive	102	12.8
	unknown	89	11.3

[a]Only female patients with primary, invasive breast cancer were included. [b]According to TNM classification by Sobin and Wittekind [1]. [c]Immunoreactive score (IRS) according to Remmele and Stegner [2]. [d]Overexpression of the *ERBB2* gene (Her-2/neu) was diagnosed analogously to the threshold of the DAKO-Score system based on IHC assay. Percentages may not sum-up to 100% due to rounding.

1. Sobin LH, Fleming ID. TNM Classification of Malignant Tumors, fifth edition (1997). Union Internationale Contre le Cancer and the American Joint Committee on Cancer. Cancer. 1997;80(9):1803-4. PubMed PMID: 9351551;

2. Remmele W, Stegner HE. Recommendation for uniform definition of an immunoreactive score (IRS) for immunohistochemical estrogen receptor detection (ER-ICA) in breast cancer tissue. Pathologe. 1987;8(3):138-40. PubMed PMID: 3303008;
