# Supplementary material for: Loss of Dickkopf 3 Promotes the Tumorigenesis of Basal Breast Cancer
Source: PLoS One. 2016 Jul 28;11(7):e0160077. doi: 10.1371/journal.pone.0160077 (PMC4965070; doi:10.1371/journal.pone.0160077)
Supplement: S5 Table — This table illustrates the sequences of all primers used in this study, the rt-PCR reaction conditions and the size of the respective amplicon. (PDF) [file pone.0160077.s005.pdf]

S5 Table: Primer sequences and PCR conditions for RNA expression analysis

Primer	Sequence	Product size [bp]
DKK3 **forward**	5’-AAGGCAGAAGGAGCCACGAGTGC-3’	182
DKK3 **reverse**	5’-GGCCATTTTGGTGCAGTGACCCCA -3’	
GAPDH **forward**	5’-GAAGGTGAAGGTCGGAGTCA-3’	108
GAPDH **reverse**	5’-AATGAAGGGGTCATTGATGG-3’	
E-Cadherin **forward**	5'-TGCCCAGAAAATGAAAAAGG-3'	200
E-Cadherin **reverse**	5'-GTGTATGTGGCAATGCGTTC-3'	
Occludin **forward**	5‘-CTATAAATCCACGCCGGTTC -3‘	243
Occludin **reverse**	5‘-GGTGGATATTCCCTGATCCA-3‘	
Claudin 1 **forward**	5‘-CTATGACCCTATGACCCCAGT -3‘	158
Claudin 1 **reverse**	5‘-TTGGATAGGGCCTTGGTGTT -3‘	
TJP1 **forward**	5‘-GCAAAAATTACAATTAGAAGGAAGAA-3‘	300
TJP1 **reverse**	5‘-TCGAAGACCATATTCTTCATTTTTC-3‘	
Snail 1 **forward**	5'-CTGGGTGCCCTCAAGATG-3'	129
Snail 1 **reverse**	5'-AGAAGGGCTTCTCGCCAGT-3'	
Vimentin **forward**	5'-TCCACGAAGAGGAAATCCAG-3'	314
Vimentin **reverse**	5'-TTCCAGGGACTCATTGGTTC-3'	

Real-time PCR reaction volumes of 20 µl consisted of the following components:

5 µM forward primer, 5 µM reverse primer, 10 µl SYBR GRN Supermix and 1 µl of cDNA as PCR template. Cycle conditions: 95°C for 3 min, 40 cycles of 95°C for 30 s, 60°C for 20 s, 72°C for 30 s. bp: base pairs.
