# Supplementary material for: Loss of Dickkopf 3 Promotes the Tumorigenesis of Basal Breast Cancer
Source: PLoS One. 2016 Jul 28;11(7):e0160077. doi: 10.1371/journal.pone.0160077 (PMC4965070; doi:10.1371/journal.pone.0160077)
Supplement: S6 Table — This table summarizes the correlation between clinico-pathological parameters of cryoconserved breast cancer samples used in this study and corresponding DKK3 mRNA expression data. (PDF) [file pone.0160077.s006.pdf]

Supplementary Table 6: Clinico-pathological parameters of cryoconserved breast cancer specimens in relation to *DKK3* mRNA expression

Parameter		n[a]	*DKK3* high[b]	*DKK3* low[b]	*P*-value[c]
Total		30	15 (50.0%)	15 (50.0%)	-
Age at diagnosis					
	<63 years	14	10 (71.4%)	4 (28.6%)	0.066
	≥63 years	16	5 (31.3%)	11 (68.7%)	
Tumor size[d]					
	pT1	14	8 (57.2%)	6 (42.8%)	0.715
	pT2-3	16	7 (43.7%)	9 (56.3%)	
Lymph node status[d]					
	pN0	17	7 (41.2%)	10 (58.2%)	0.462
	pN1-3	12	7 (58.3%)	5 (41.7%)	
Histological tumor grade[e]					
	G1-2	8	7 (87.5%)	1 (12.5%)	**0.035**
	G3	22	8 (36.4%)	14 (63.6%)	
Oestrogen receptor status					
	negative (IRS[f] 0-2)	16	4 (25.0%)	12 (75.0%)	**0.009**
	positive (IRS[f] 3-12)	14	11 (78.6%)	3 (21.4%)	
Progesterone receptor status					
	negative (IRS[f] 0-2)	15	5 (33.3%)	10 (66.7%)	0.066
	positive (IRS[f] 3-12)	15	10 (66.7%)	5 (33.3%)	
HER2 status[g]					
	negative	24	11 (45.8%)	13 (54.2%)	0.651
	positive	6	4 (66.7%)	2 (33.3%)	

[a]Only female patients with primary, unilateral, invasive breast cancer were included. [b]Expression was dichotomized into "*DKK3* high" and "*DKK3* low" based on the median *DKK3* expression. [c]Fisher's exact test. Significant P-values are marked in bold face. [d]According to TNM classification by Sobin and Wittekind [1]. [e]According to Bloom and Richardson, as modified by Elston and Ellis [2]. [f]Immunoreactive score (IRS) according to Remmele and Stegner [3]. [g]Overexpression of the *ERBB2* gene (Her-2/neu) was diagnosed analogously to the threshold of the DAKO-Score system based on IHC assay. Uncertain cases were additionally validated by FISH assay. Percentages may not sum-up to 100% due to rounding.

1. Sobin LH, Fleming ID. TNM Classification of Malignant Tumors, fifth edition (1997). Union Internationale Contre le Cancer and the American Joint Committee on Cancer. Cancer. 1997;80(9):1803-4. PubMed PMID: 9351551;

2. Elston CW, Ellis IO. Pathological prognostic factors in breast cancer. I. The value of histological grade in breast cancer: experience from a large study with long-term follow-up. Histopathology. 1991;19(5):403-10. PubMed PMID: 1757079;

3. Remmele W, Stegner HE. Recommendation for uniform definition of an immunoreactive score (IRS) for immunohistochemical estrogen receptor detection (ER-ICA) in breast cancer tissue. Pathologe. 1987;8(3):138-40. PubMed PMID: 3303008;
