# Supplementary material for: Loss of Dickkopf 3 Promotes the Tumorigenesis of Basal Breast Cancer
Source: PLoS One. 2016 Jul 28;11(7):e0160077. doi: 10.1371/journal.pone.0160077 (PMC4965070; doi:10.1371/journal.pone.0160077)
Supplement: S7 Table — This table summarizes the correlation between clinico-pathological parameters of TCGA in silico breast cancer samples used in this study and corresponding DKK3 mRNA expression data. (PDF) [file pone.0160077.s007.pdf]

Supplementary Table 7: Clinico-pathological parameters of breast cancer specimens obtained from *The Cancer Genome Atlas* platform in relation to *DKK3* mRNA expression

**Parameter**		**n[a]**	***DKK3* high[b]**	***DKK3* low[b]**	***P*-value[c]**
Total		791	401 (50.7%)	390 (49.3%)	-
Age at diagnosis					
	≤58 years	409	213 (52.1%)	196 (47.9%)	0.434
	>58 years	382	188 (49.2%)	194 (50.8%)	
Tumor size[d]					
	pT1	209	123 (58.9%)	86 (41.2%)	**0.006**
	pT2-4	582	278 (47.8%)	304 (52.2%)	
Lymph node status[d]					
	pN0	375	178 (47.5%)	197 (52.5%)	0.073
	pN1-3	403	218 (54.1%)	185 (45.9%)	
Estrogen receptor status					
	negative (IRS[e] 0-2)	176	61 (34.7%)	115 (65.3%)	**>0.001**
	positive (IRS[e] 3-12)	577	321 (55.6%)	256 (44.4%)	
Progesterone receptor status					
	negative (IRS[e] 0-2)	246	96 (39.0%)	150 (61.0%)	**>0.001**
	positive (IRS[e] 3-12)	504	285 (56.6%)	219 (43.5%)	
HER2 status[f]					
	negative	600	296 (49.3%)	304 (50.7%)	**0.041**
	positive	102	62 (60.8%)	40 (39.2%)	

[a]Only female patients with primary, invasive breast cancer were included. [b]Expression was dichotomized into "*DKK3* high" and "*DKK3* low" based on the median *DKK3* expression. [c]Fisher's exact test. Significant P-values are marked in bold face. [d]According to TNM classification by Sobin and Wittekind [1]. [e]Immunoreactive score (IRS) according to Remmele and Stegner [2]. [f]Overexpression of the *ERBB2* gene (Her-2/neu) was diagnosed analogously to the threshold of the DAKO-Score system based on IHC assay. Percentages may not sum-up to 100% due to rounding.

1. Sobin LH, Fleming ID. TNM Classification of Malignant Tumors, fifth edition (1997). Union Internationale Contre le Cancer and the American Joint Committee on Cancer. Cancer. 1997;80(9):1803-4. PubMed PMID: 9351551;

2. Remmele W, Stegner HE. Recommendation for uniform definition of an immunoreactive score (IRS) for immunohistochemical estrogen receptor detection (ER-ICA) in breast cancer tissue. Pathologe. 1987;8(3):138-40. PubMed PMID: 3303008;
